# Hysteresis Responses of Evapotranspiration to Meteorological Factors at a Diel Timescale: Patterns and Causes

**DOI:** 10.1371/journal.pone.0098857

**Published:** 2014-06-04

**Authors:** Han Zheng, Qiufeng Wang, Xianjin Zhu, Yingnian Li, Guirui Yu

**Affiliations:** 1 Synthesis Research Center of Chinese Ecosystem Research Network, Key Laboratory of Ecosystem Network Observation and Modeling, Institute of Geographic Sciences and Natural Resources Research, Chinese Academy of Sciences, Beijing, China; 2 Northwest Institute of Plateau Biology, Chinese Academy of Sciences, Xining, China; 3 University of Chinese Academy of Sciences, Beijing, China; The Ohio State University, United States of America

## Abstract

Evapotranspiration (ET) is an important component of the water cycle in terrestrial ecosystems. Understanding the ways in which ET changes with meteorological factors is central to a better understanding of ecological and hydrological processes. In this study, we used eddy covariance measurements of ET from a typical alpine shrubland meadow ecosystem in China to investigate the hysteresis response of ET to environmental variables including air temperature (*T*_a_), vapor pressure deficit (VPD) and net radiation (*R*_n_) at a diel timescale. Meanwhile, the simulated ET by Priestly-Taylor equation was used to interpret the measured ET under well-watered conditions. Pronounced hysteresis was observed in both *T*_a_ and VPD response curves of ET. At a similar *T*_a_ and VPD, ET was always significantly depressed in the afternoon compared with the morning. But the hysteresis response of ET to *R*_n_ was not evident. Similar hysteresis patterns were also observed in the *T*_a_/VPD response curves of simulated ET. The magnitudes of the measured and simulated hysteresis loops showed similar seasonal variation, with relatively smaller values occurring from May to September, which agreed well with the lifetime of plants and the period of rainy season at this site. About 62% and 23% of changes in the strength of measured ET-*T*_a_ and ET-VPD loops could be explained by the changes in the strength of simulated loops, respectively. Thus, the time lag between *R*_n_ and *T*_a_/VPD is the most important factor generating and modulating the ET-*T*_a_/VPD hysteresis, but plants and water status also contribute to the hysteresis response of ET. Our research confirmed the different hysteresis in the responses of ET to meteorological factors and proved the vital role of *R*_n_ in driving the diel course of ET.

## Introduction

Evapotranspiration (ET), an important component of the water cycle and the principal route of water loss in terrestrial ecosystems, strongly affects biogeochemical cycles and the surface energy balance and further influences the local weather and climate [1], [2]. A number of environmental variables (e.g., net radiation, vapor pressure deficit, air temperature, wind speed and soil water content) have significant impacts on the variability of ET, whereas the dominant factor varies at different spatiotemporal scales [2]–[7]. Therefore, analyzing the responses of ET to environmental variables is of great significance to achieve a better understanding of the water cycle.

Hysteresis between transpiration (or sap flow) and microclimate variables (mainly VPD) has been widely reported on the basis of individual plants or even more small units (e.g., tree branches) [8]–[15]. As we know, ET is generally parameterized as the sum of vegetation transpiration and soil evaporation [2], [16]. Accordingly, the response patterns of ET to environmental factors are the integrated consequences of all processes and parameters influencing transpiration and evaporation. The dominance of transpiration in terrestrial ET [17] suggests that hysteresis might occur in responses of ET to environmental factors. To our knowledge, however, only a few studies [18]–[20] reported the hysteresis phenomenon in the response of ET to photosynthetically active radiation (PAR) or VPD. As these results were based on quite short-term data and only one environmental factor was considered, the consistency of hysteresis at a long-term scale remains to be questioned and more environmental factors should be taken into account if a more complete understanding of the ecosystem water cycle and its response mechanisms to environmental variables is desired.

Alpine meadows are characterized by relatively high precipitation, strong solar radiation and low temperature [21] and are very sensitive and vulnerable to global climate change [22], providing a special opportunity to examine ecosystem ET in response to changes in environmental conditions. The eddy covariance (EC) method continuously monitors water flux between the ecosystem and atmosphere and associated environmental factors, facilitating the analysis of the response patterns of ET to environmental factors [23]. In the present study, we used the EC flux measurements from a typical alpine shrubland meadow ecosystem, a component of the Chinese Terrestrial Ecosystem Flux Research Network (ChinaFLUX), during 2003–2011 to investigate the diel course of ET and its response patterns to key environmental factors including air temperature (*T*_a_), vapor pressure deficit (VPD) and net radiation (*R*_n_) at a diel timescale. This research seeks to answer the following scientific questions: (1) Is hysteresis observed in the response of ET to environmental factors at a diel timescale (24 h)? (2) To which environmental factor(s) does ET respond with strong hysteresis? (3) What are the seasonal patterns of hysteresis? and (4) What are the potential causes of hysteresis and of its seasonal variation?

## Materials and Methods

### Site Description

The study site is a component of the ChinaFLUX network and is classified as an alpine shrubland meadow. It is located at the Haibei Research Station, Chinese Academy of Sciences, in Qinghai Province, China (latitude 37°39′55″N, longitude 101°19′52″E, altitude 3293 m). The site is located on the northeastern Qinghai-Tibetan Plateau and experiences a typical plateau continental climate with long, cold winters and short, cool summers. Climate data show that the annual average air temperature and amount of annual precipitation are −1.6°C and 560 mm, respectively (data from 1980 to 2001 from the Haibei climate station). Approximately 80% of the annual rainfall falls within the rainy season, which is also the growing season, extending from May to September [24]. The soil type is Mollic-Cryic Cambisols [25], with rich organic content and a thin soil layer. The vegetation is dominated by *Dasiphora fruticosa* and reaches a height of 30–40 cm. Many herbaceous plants with mean heights of 8–20 cm grow in the meadow; the dominant species are *Helictotrichon tibeticum* and *Elymus nutans*. The vegetation at the study site starts to green at the end of April or early May and begins to senesce in late September or early October depending on the meteorological conditions of the given year. More detailed site information is available in previous studies [25].

### Measurements

Water vapor flux and micrometeorological conditions were measured since September 2002. The EC system was composed of a three-dimensional sonic anemometer (CSAT3, Campbell, USA) and an open-path fast response infrared CO_2_/H_2_O analyzer (Li-7500, Li-Cor Inc, USA). The sensors were mounted at a height of 2.2 m above the ground with a fetch of more than 250 m in all directions [26]. The EC measurements were sampled with a frequency of 10 Hz and then recorded with a data logger at 30-min intervals. Meteorological variables, including *R*_n_, *T*_a_, incident solar radiation (*R*_s_) and relative humidity (RH), were measured and calculated simultaneously at 30-min intervals. VPD was calculated from the observations of *T*_a_ and RH. Further details of the monitoring system were presented by Zhao *et al*. (2005) [26]. The energy closure ratio of this site exceeded 0.7 [27], indicating reasonable data quality.

### Data Processing

To process the raw 30-min flux data, we applied the routine processing procedures recommended by ChinaFLUX, including three-dimensional coordinate rotation, WPL (Webb-Pearman-Leuning) correction, removal of anomalous values and gap filling [28]. The three-dimensional rotation was applied to make the average vertical wind speed to zero and to force the horizontal wind to the mean wind direction. WPL correction was used to adjust the effect of density change on CO_2_ and H_2_O fluxes (Webb *et al.*, 1980) [29].

Spurious data caused by rainfall, water condensation or system failure were removed from the dataset. To exclude the low turbulence fluxes at night, the friction velocity (u*) threshold was calculated according to Reichstein et al (2005) [30] as follows: Firstly, every three months data were grouped into one set and each year data were divided into four sets. Secondly, in each set, all available data were split into six subsets having the same sample size and the similar temperature. In each subset, data were divided into 20 classes by u*. The threshold of u* in this subset was defined as the u* value where the carbon flux was higher than the 95% of the average flux at the higher u* class. The threshold of u* for each set was calculated as the median of the u* threshold from six subsets. Then the u* threshold for the whole dataset was set as the highest threshold found.

Data gaps due to missing or rejected data are unavoidable in long-term and continuous measurements. We filled short gaps (<2 h) in the water flux data with linear interpolation, and we applied a look-up table method for the longer gaps [30], [31] using *T*_a_, VPD and *R*_s_. For the meteorological data, we used linear interpolation for gaps of less than 2 h and the mean diurnal variation method for the longer gaps [31]. All the data processing procedures specified above were performed with a Matlab program written in-house.

### ET Calculated by Priestly-Taylor Equation

To assess the effects of time lag between *R*_n_ and *T*_a_/VPD on the ET-*T*_a_/VPD hysteresis, Priestly-Taylor (P-T) equation, which is a simple ET model driven by only *R*_n_ and *T*_a_, was used in this study. Under well-watered conditions, P-T equation can be written as [2], [32]:
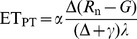
where ET_PT_, the ET calculated by P-T equation (gH_2_O m^−2^ s^−1^), could be used to interpret eddy covariance measurements in well watered conditions; α, the Priestly-Taylor coefficient, is given as 1.26 for wet or humid conditions in this study; Δ is the slope of relation between saturation vapor pressure and temperature (kPa °C^−1^); γ is the psychrometric constant (kPa °C^−1^); *R*_n_ and G are net radiation and soil heat flux (W m^−2^), respectively; and λ is the latent heat of vaporization (2.45 MJ kg^−1^). When nighttime PAR was smaller than 10 µmol photon m^−2^s^−1^ or the P-T model prediction was negative, the P-T simulations for ET were set to zero in this study.

### Data Analyses

Monthly averaged 30-min values of ET and relevant meteorological variables (including *T*_a_, VPD and *R*_n_) collected from 2003 to 2011 were analyzed. The monthly averaged values were calculated by averaging each 30-min datum for a particular time over 1 month. ET was then plotted against each of the corresponding *T*_a_, VPD and *R*_n_ datasets. Monthly averaged 30-min data for ET, *T*_a_, VPD and *R*_n_ from 2009 to 2011 are available in Table S1.

All the response curves plots specified above consisted of two parts: an increasing part, in which ET increased with changes in *T*_a_/VPD/*R*_n_, and a decreasing part, in which ET decreased with changes in *T*_a_/VPD/*R*_n_. In this study, we defined the increasing part as ‘morning’ and the decreasing part as ‘afternoon’. We used a t-test to analyze the significance of the hysteretic relationship between the morning and afternoon during one day. The difference was considered significant if *P*<0.05.

To illustrate the month-to-month variation in hysteresis, normalized plots were used to remove the influence of the magnitudes of ET and *T*_a_/VPD from the data. In a normalized plot, the proportion of maximum ET of the specific month was plotted against the proportion of maximum *T*_a_ or the proportion of maximum VPD. Here, *T*_a_ is absolute temperature converted by the measured centigrade temperature by adding 273.15. Then the area within the normalized hysteresis loop (H_area_) was computed with a built-in function named *Polyarea* in Matlab R2009a to quantify the strength of hysteresis. The normalized plots were also used to compare the differences of ET-VPD (and ET-*T*_a_) hysteresis between the EC measurements and P-T calculations. A simple regression was used to analyze the relationship between the EC H_area_ and P-T H_area_, which was considered significant if *P*<0.05. All the statistical analyses were performed with SPSS 16.0 for Windows, and all figures were plotted using Matlab R2009a.

## Results

### Diel Patterns of ET and Meteorological Factors

**Figure 1:**
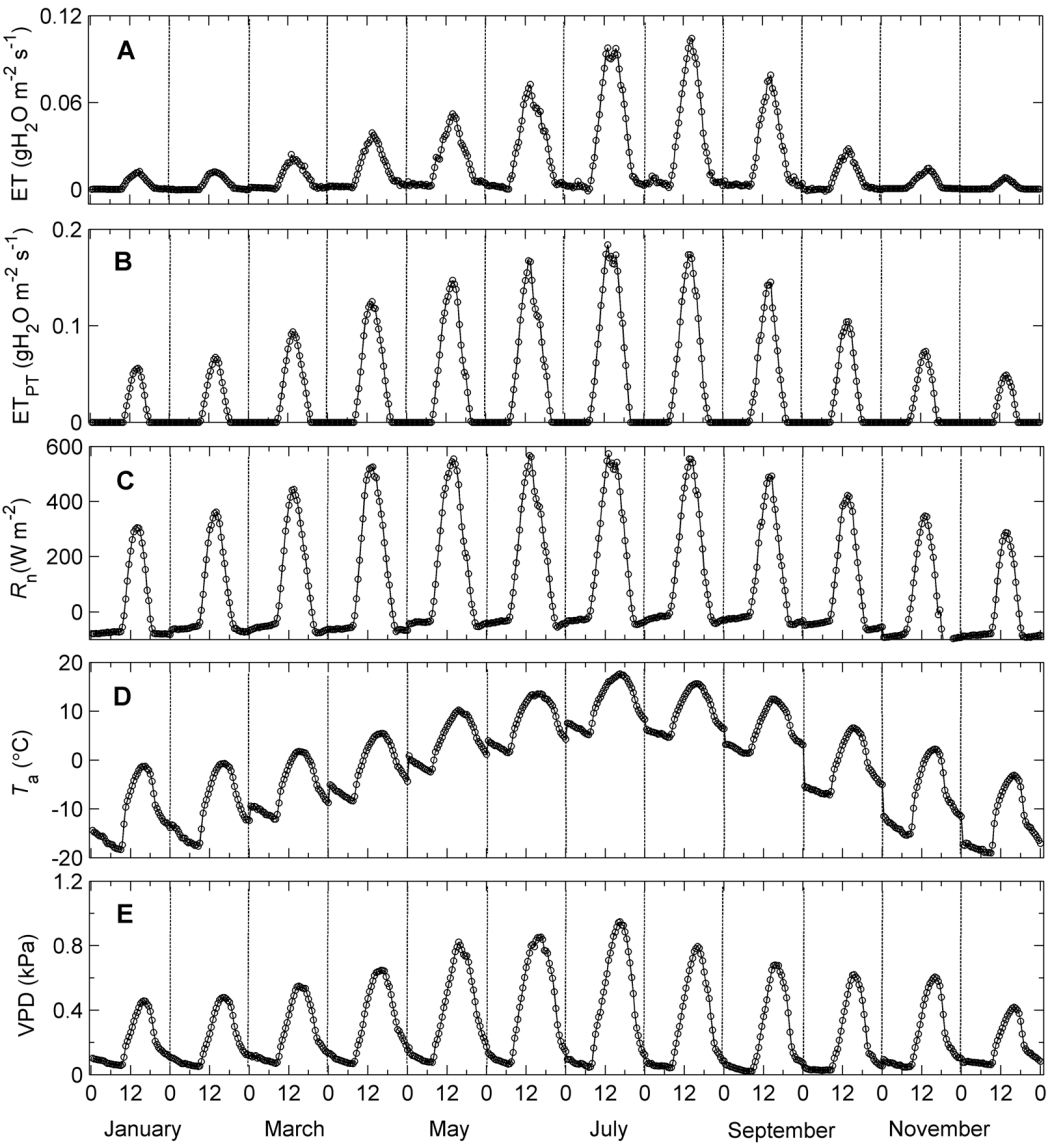
Diel patterns of evapotranspiration (ET) measured by eddy covariance method (A), calculated ET using Priestly-Taylor equation (ET_PT_, B), net radiation (*R*_n_, C), air temperature (*T*_a_, D), and vapor pressure deficit (VPD, E). Data points are half-hourly values averaged monthly during 2010. BST is depicted in the upper *x axis* (0 =  midnight, 12 =  noon), and month is depicted in the lower *x axis*.

The monthly averaged diel patterns of measured ET by eddy covariance method and model-calculated ET using Priestly-Taylor equation (ET_PT_) were markedly unimodal during almost every month (Figure 1A and 1B). The ET values during the night were low and showed small changes, then rapidly increased and peaked at approximately 13∶00–14∶00 Beijing Standard Time (BST, approximately 1 h earlier than the solar time at the study site). ET then began to decline and approached zero at approximately 18∶00–20∶00 BST (depending on the season). *R*_n_, VPD and *T*_a_ also presented similar diel trends (Figure 1C, 1D and 1E) and *R*_n_ was almost in phase with both measured and simulated ET. *R*_n_ attained its peak values at approximately the same time as ET, whereas the peak values of *T*_a_ and VPD lagged well behind ET for almost 2 h.

### The Relationship between ET and Meteorological Factors

**Figure 2:**
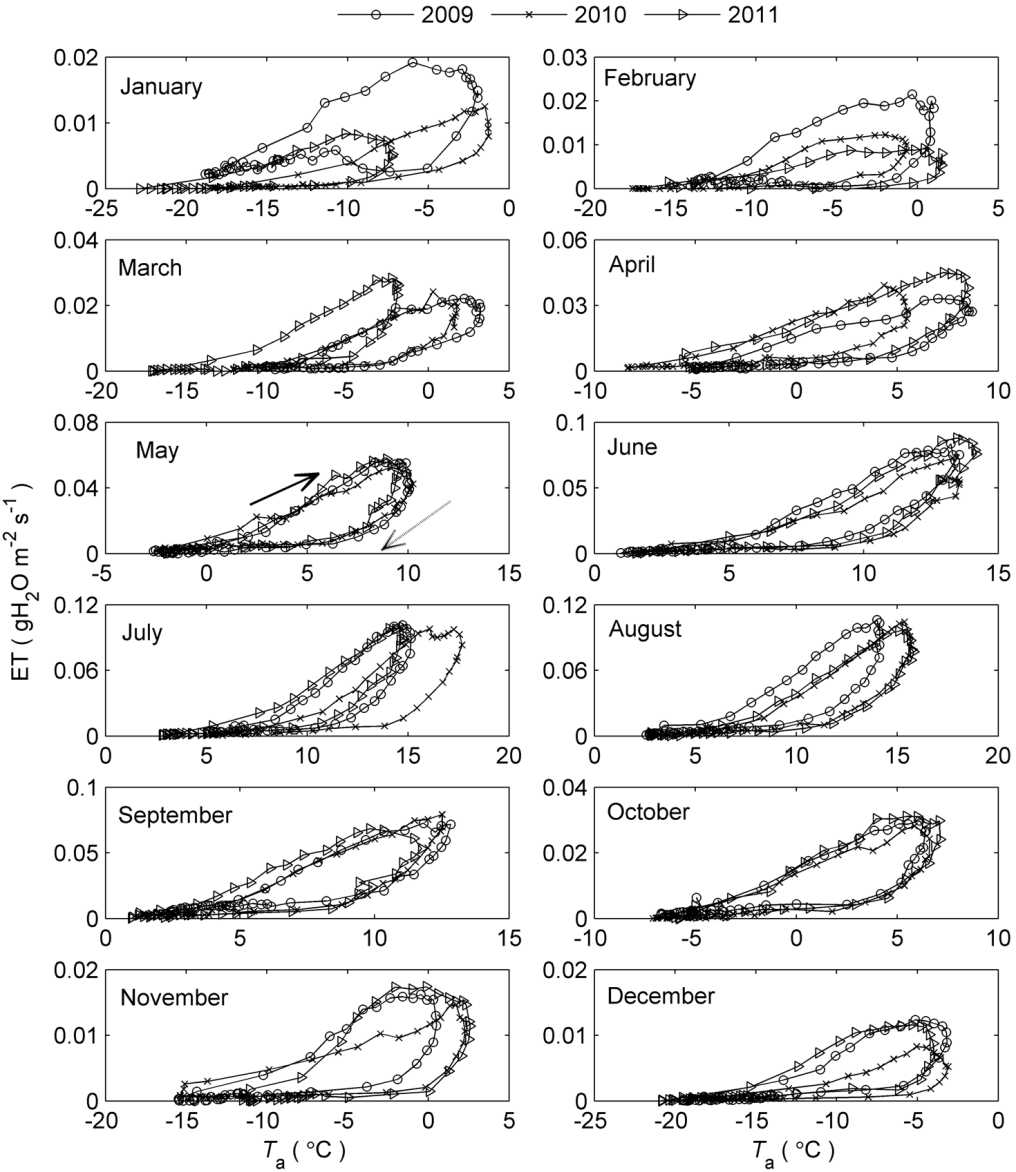
Temperature response curves of ET at diel timescale. Data points are half-hourly values averaged monthly. The solid arrows indicate the direction of response in the morning and the dashed ones indicate the direction of response in the afternoon. The area enclosed by the ET trajectories measures the strength of the hysteresis loop.

**Figure 3:**
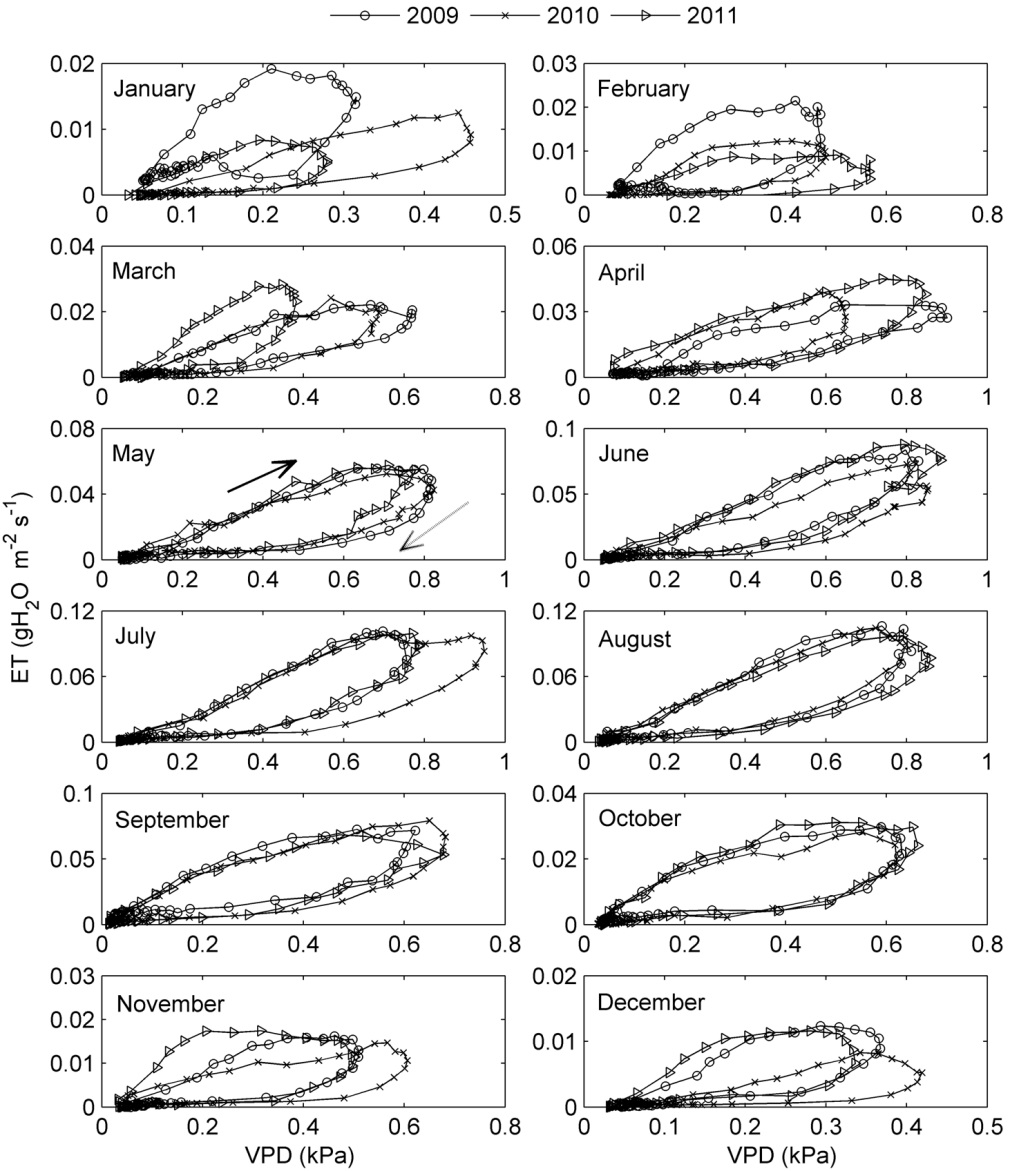
Vapor pressure deficit response curves of ET at diel timescale. Data points are half-hourly values averaged monthly. The solid arrows indicate the direction of response in the morning and the dashed ones indicate the direction of response in the afternoon. The area enclosed by the ET trajectories measures the strength of the hysteresis loop.

**Figure 4:**
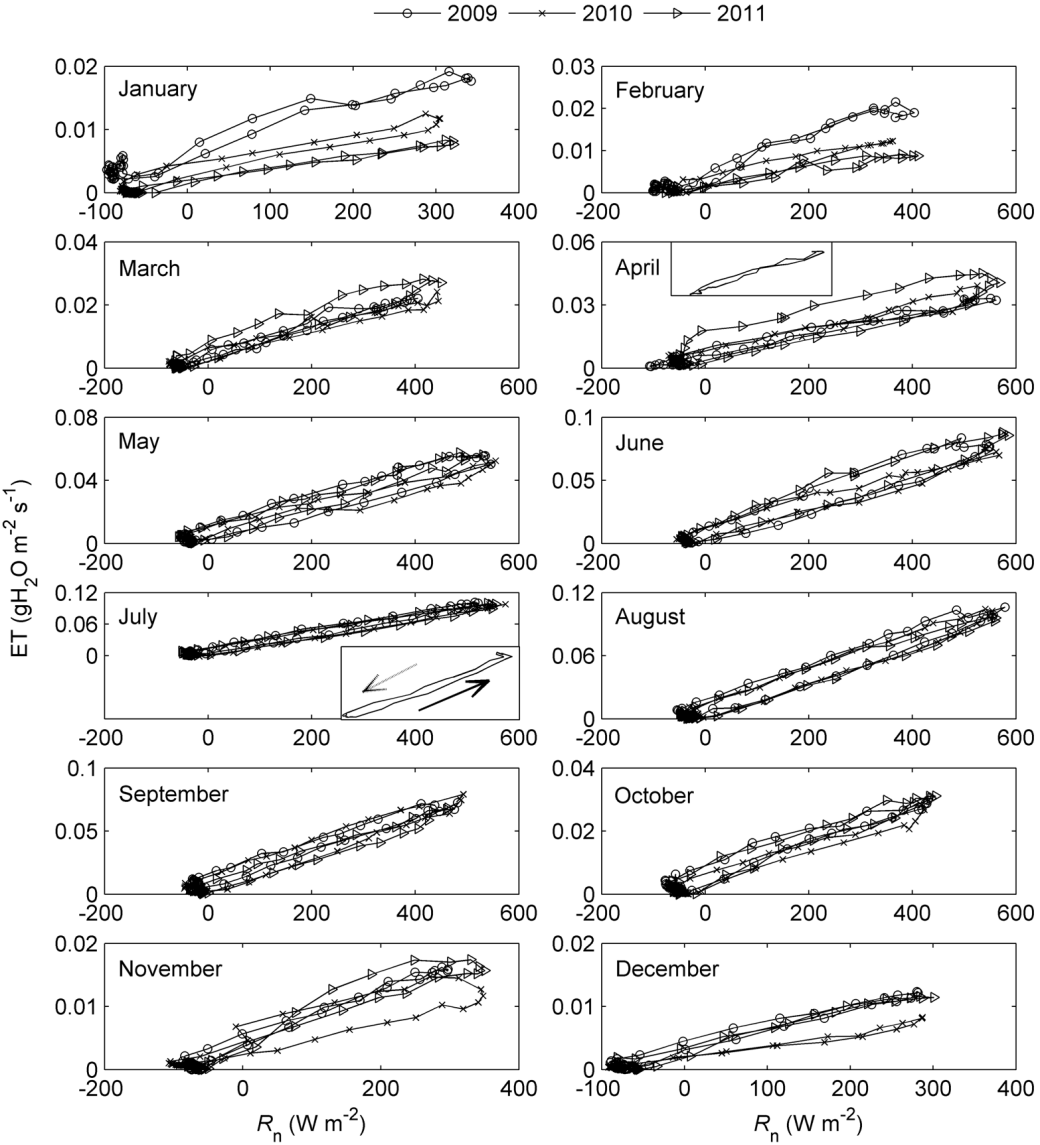
Net radiation response curves of ET at diel timescale. Data points are half-hourly values averaged monthly. The thumbnails in subplots ‘April’ and ‘July’ are *R*_n_ response curves in April of 2009 and July of 2010, respectively. The solid arrow indicates the direction of response in the morning and the dashed one indicates the direction of response in the afternoon.

Distinct clockwise hysteresis loops were observed in the relationship between ET and *T*_a_ for all diel cycles averaged monthly from 2003 to 2011 (Figure 2, Figure S1 and Figure S2). As *T*_a_ increased in the morning, ET increased and peaked at an optimum temperature. ET then declined with decreasing *T*_a_ in the afternoon. However, at the same temperature, ET was always higher in the morning than in the afternoon, forming a significant clockwise hysteresis loop (*P*<0.05 for all months except October 2007). Obvious clockwise hysteresis loops were also observed in the VPD response curves of ET (Figure 3, Figure S3 and Figure S4). Specifically, the morning values of ET were greater than the afternoon values of ET at any given value of VPD (*P*<0.05 for all months except October 2007). The trend in the variation of the *R*_n_ response curves of ET was similar to that of the *T*_a_ and VPD response curves (Figure 4, Figure S5 and Figure S6), with anti-clockwise loops observed for many months (e.g., Figure 4-July). However, the hysteresis was much weaker than that of the ET-*T*_a_ and ET-VPD relationships (*P*>0.05 for all months except April 2009 and July 2010). The patterns of the incident solar radiation (*R*_s_) response curves were similar to those of the *R*_n_ response curves, with no significant hysteresis detected (figure not shown).

**Figure 5:**
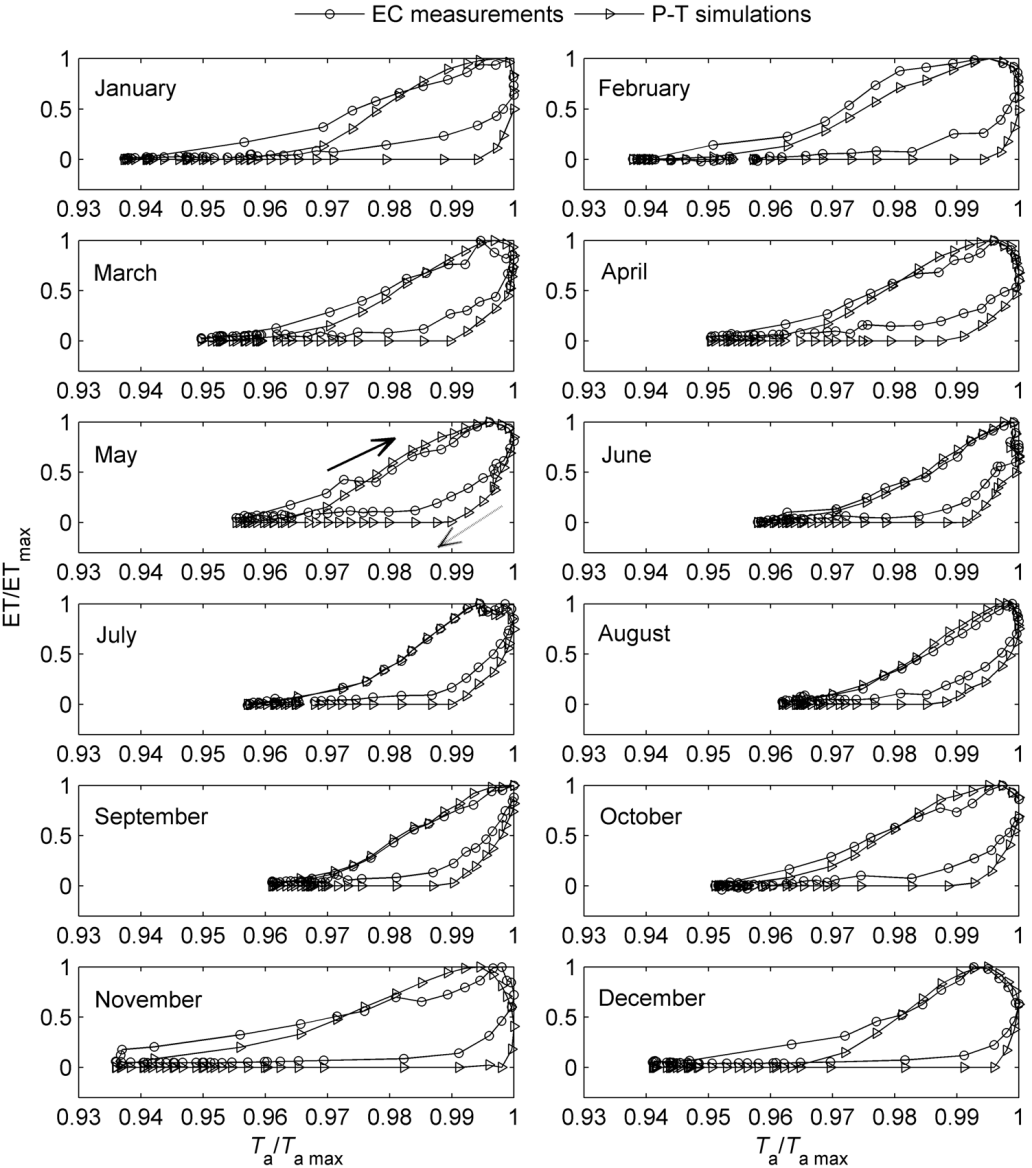
An example of seasonal variation in ET-*T*_a_ hysteresis using normalized plots in 2010. The *y axis* represents the proportion of maximum ET (dimensionless) and the *x axis* represents the proportion of maximum *T*_a_ (dimensionless). Here, *T*_a_ is absolute temperature (K) converted by the measured centigrade temperature (°C) by adding 273.15. The solid arrow indicates the direction of response in the morning and the dashed one indicates the direction of response in the afternoon. The area enclosed by the ET trajectories measures the strength of the hysteresis loop.

**Figure 6:**
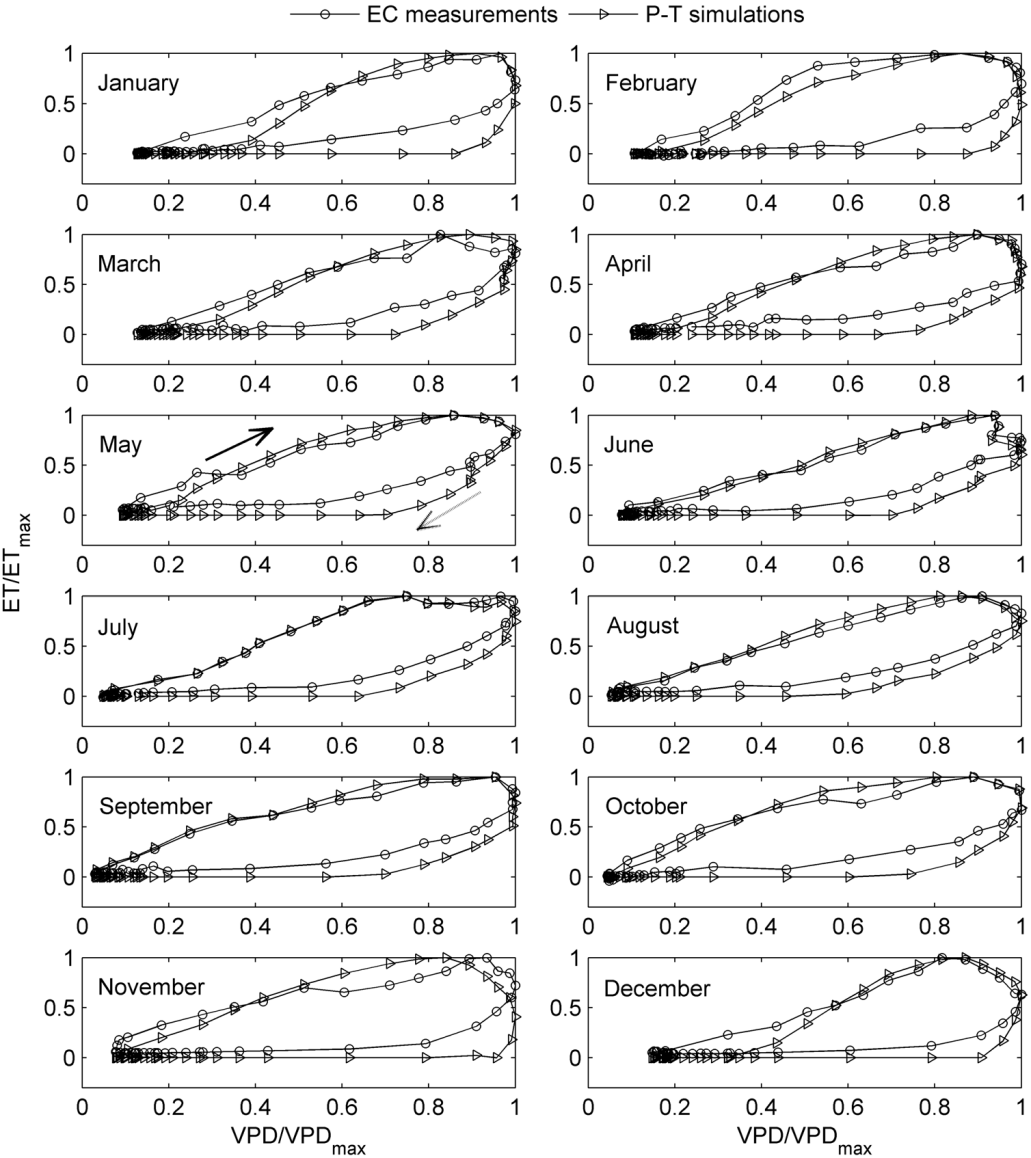
An example of seasonal variation in ET-*T*_a_ hysteresis using normalized plots in 2010. The *y axis* represents the proportion of maximum ET (dimensionless) and the *x axis* represents the proportion of maximum VPD (dimensionless).The solid arrow indicates the direction of response in the morning and the dashed one indicates the direction of response in the afternoon. The area enclosed by the ET trajectories measures the strength of the hysteresis loop.

All the *T*_a_/VPD/*R*_n_ response curves of simulated ET using P-T equation also exhibited similar trend with the corresponding curves of the measured ET, i.e., pronounced ET-*T*_a_ and ET-VPD hysteresis (Figure 5 and Figure 6) while no distinct hysteresis in ET-*R*_n_ relationship (Figure not shown). Moreover, most of the time, the simulated loops were larger than the measured ones, however, their upper parts (i.e., in the morning) nearly overlapped during many months (Figure 5 and Figure 6).

### Seasonal Patterns of ET-*T*_a_ and ET-VPD Hysteresis

**Figure 7:**
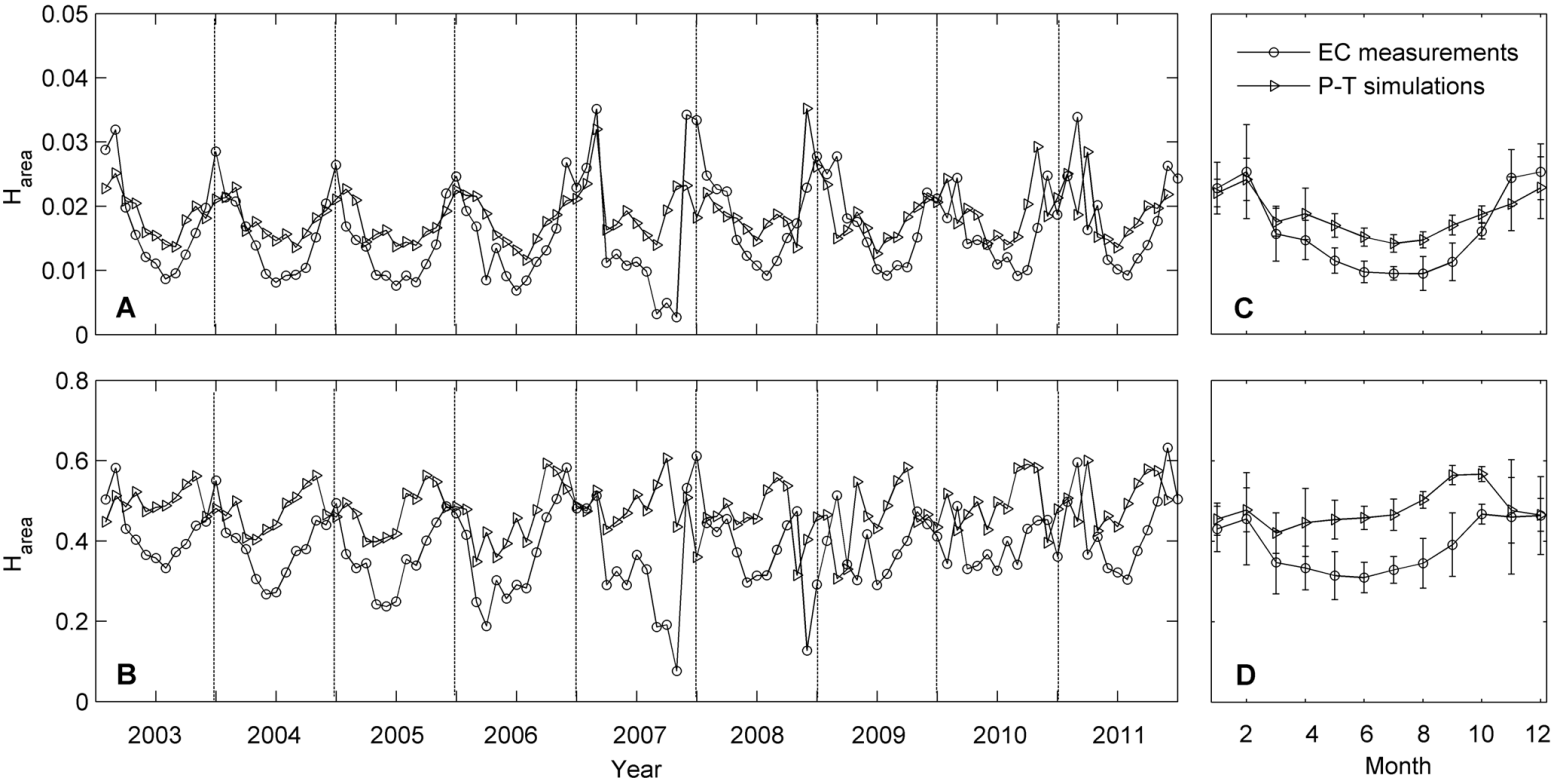
Seasonal variation in ET-*T*_a_ (A and C) and ET-VPD (B and D) hysteresis area from 2003 to 2011. Each data point in the left panels represents the hysteresis area (H_area_) for a month, while each data point in the right panels represents a multi-year average of H_area_ (mean ± 1SE) for a particular month from 2003 to 2011 (datum in October 2007 was eliminated). H_area_ is calculated from normalized plot (e.g., Figure 5 and Figure 6).

Although the clockwise hysteresis pattern was highly consistent in both the *T*_a_ and VPD response curves of ET, the degree of the hysteresis loops differed substantially among the months during the year. An example of the seasonal variation in ET-*T*_a_ and ET-VPD hysteresis is presented in Figure 5 and Figure 6, respectively, using normalized plots to remove the influence of the magnitudes of ET and *T*_a_/VPD from the data. There was pronounced month-to-month variation in hysteresis, especially in ET-*T*_a_ hysteresis (Figure 5). The seasonal patterns of ET hysteresis were quite similar during the nine years, though subtle differences existed (Figure 7). In general, both the magnitudes of the ET-*T*_a_ and ET-VPD hysteresis (manifested by the area within the hysteresis loop, H_area_) were relatively small from May to September (Figure 7).

**Figure 8:**
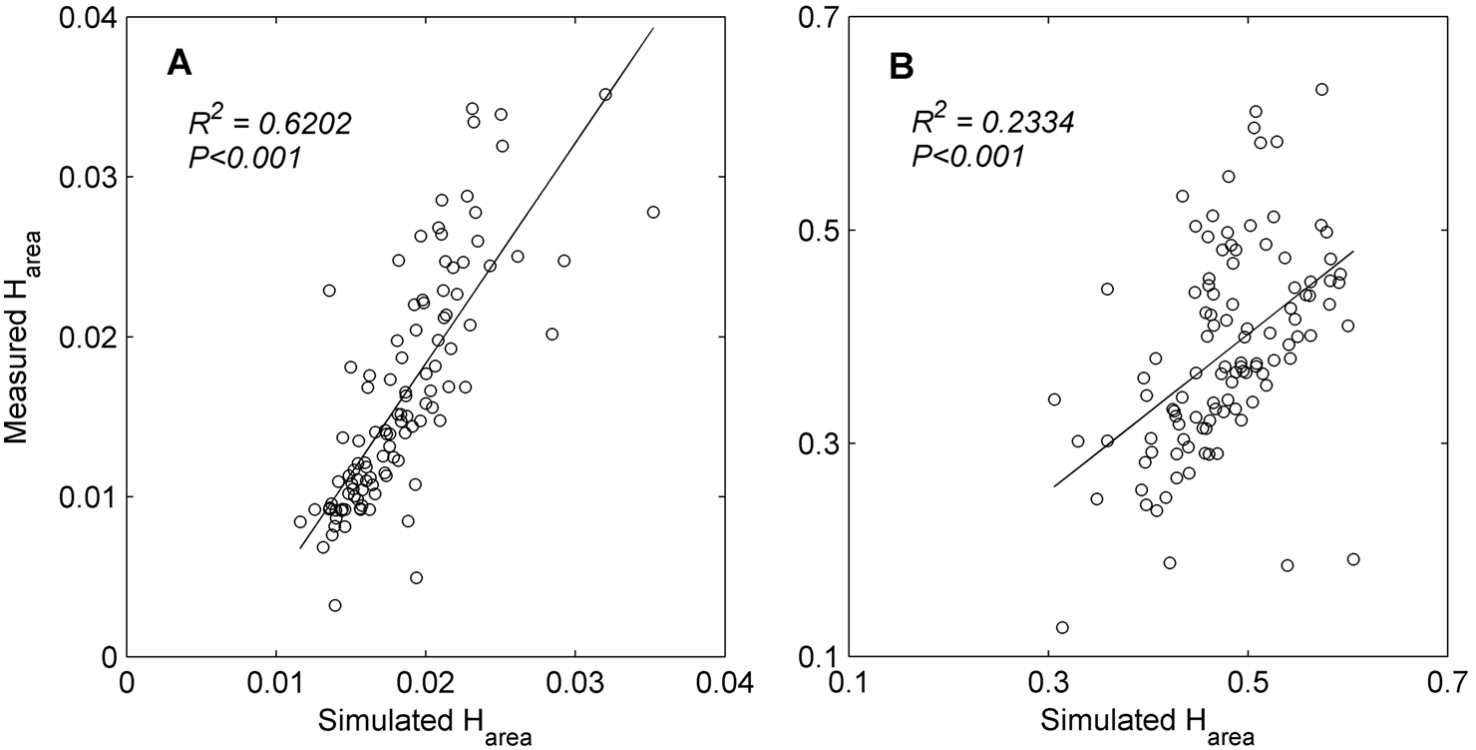
Relationship between the area within the measured and simulated hysteresis loops. Panel A and B are with respect to ET-*T*_a_ and ET-VPD relation, respectively. H_area_ is calculated from normalized plot (e.g., Figure 5 and Figure 6).

At the meantime, the patterns of variation in the simulated ET hysteresis by P-T equation agreed very well with that of the measured hysteresis in both VPD and *T*_a_ situations among the observation period (Figure 7). Simple regression analyses demonstrated that H_area_ of simulated ET-*T*_a_ and ET-VPD loops could explain about 62% and 23% of changes in that of measured loops, respectively (Figure 8). Meanwhile, the simulated H_area_ was virtually always bigger than the measured H_area_, but the gaps between simulated H_area_ and measured H_area_ were not nearly equal through the year, but with relatively larger gaps from May to September (Figure 7).

## Discussion

### Hysteresis in ET-*T*_a_ and ET-VPD Relationships

In this study, diel patterns of ET were almost in phase with that of *R*_n_, while phase differences existed between *T*_a_/VPD and *R*_n_ (Figure 1). Thus, the time lag between *R*_n_ and *T*_a_/VPD might be an important factor generating the ET-VPD hysteresis, because *R*_n_ is the primary driver for evaporation. To assess this, simulated ET by Priestly-Taylor equation for a well-watered surface was used to remove the influence of real water status of plants. It turned out that the looping patterns showed up in both the measured and calculated ET-*T*_a_/VPD relationships (Figure 5 and Figure 6), which demonstrated the vital role of the time lag between *R*_n_ and *T*_a_/VPD in forming the ET-*T*_a_/VPD hysteresis loops.

Since the role of plant water status of the study site has not been considered in the P-T equation, the measured and calculated ET-*T*_a_/VPD hysteresis loops were not exactly the same in our research. For example, overlaps were observed between the measured and simulated hysteresis loops in the morning (Figure 5 and Figure 6). This is because the water supply capacity of the plants and even the soil in the morning is larger than that in the afternoon. In the morning, water may even be secreted from the hydathodes at the top or margin of the leaves [33] following rehydration at night. In contrast, plants may be partially dehydrated in the afternoon [9], [12], [14]. Dew appearing on exposed surfaces of the plants and the soil in the morning or evening due to condensation will also most likely contribute to increase the water supply capacity and hence produce a higher ET in the morning than in the afternoon.

Besides, the opening/closing behavior of stomata might also contribute to the emergence of hysteresis. Previous studies found that stomata responded differently to changes in environmental factors (e. g., VPD) during the processes of stomatal opening and closing, with larger stomatal conductance in the morning [34], [35], indicating higher canopy conductance [36] and hence higher ET in the morning compared with the afternoon.

### Seasonal Variation in ET-*T*_a_ and ET-VPD Hysteresis

Pronounced seasonal variation has been observed in both the measured and simulated ET hysteresis in our study, with relatively small magnitudes (manifested by the area within the hysteresis loop, H_area_) from May to September (Figure 7). As indicated by our simple regression analyses, changes in the simulated H_area_ of simulated ET-*T*_a_ and ET-VPD loops could explain approximately 62% and 23% of changes in that of measured loops, respectively (Figure 8). That’s to say, the variation in the phase difference (or time lag) between *R*_n_ and *T*_a_/VPD is an important factor modulating the strength of ET-*T*_a_/VPD hysteresis, though it is not clear yet why the relation between the magnitudes of simulated and measured loops is stronger in the ET-*T*_a_ relationship.

Gaps existed between the simulated and measured curves of H_area_, which is easy to understand. However, the gaps were not nearly equal through the year, but with relatively larger gaps from May to September, which agrees well with the lifetime of plants and also the period of rainy season at this study site. Hence, we speculate that the development of canopy and the water status of this ecosystem might have weakened the degree of ET hysteresis during the large-gap period, i.e., from May to September. But it is still unclear how plants and water status regulate the strength of hysteresis, which may be deeply investigated in future.

### Hysteresis Responses of ET to Meteorological Factors

Pronounced clockwise hysteresis loops were consistently rather than accidentally observed in the ET-*T*_a_ and ET-VPD relationships at a diel timescale based on the nine-year observations of the alpine shrubland meadow ecosystem (Figure 2 and Figure 3). This hysteresis phenomenon of ET was similar to that of transpiration in response to VPD, as documented in several previous studies (e.g., [10], [12], [13], [15]). These results indicate that the diel patterns of ET in response to VPD and *T*_a_ agree well with the diel response patterns of vegetation transpiration. Similarly, several researchers have also observed diel hysteresis patterns between transpiration and *R*_s_ (or PAR) [8], [10], [11], [15], with loops that were anti-clockwise [10] or in totally opposite directions before and after rainfall [15]. However, the diel hysteresis response between ET and *R*_n_ found in this study was not significant, although small anti-clockwise loops occurred frequently (Figure 4). This result might illustrate the differences between the characteristics of the responses of ET and transpiration to solar radiation.

The hysteresis responses of ET to *T*_a_/VPD indicate that using *T*_a_ or VPD to simply simulate ET may induce certain uncertainties, while we can conservatively believe that based on the *R*_n_ data, a simple model can be obtained to simulate the diel variation of ET.

## Supporting Information

Figure S1 **Temperature response curves of ET at diel timescale from 2003 to 2005.**
(TIF)

Figure S2 **Temperature response curves of ET at diel timescale from 2006 to 2008.**
(TIF)

Figure S3 **Vapor pressure deficit response curves of ET at diel timescale from 2003 to 2005.**
(TIF)

Figure S4 **Vapor pressure deficit response curves of ET at diel timescale from 2006 to 2008.**
(TIF)

Figure S5 **Net radiation response curves of ET at diel timescale from 2003 to 2005.**
(TIF)

Figure S6 **Net radiation response curves of ET at diel timescale from 2006 to 2008.**
(TIF)

Table S1 **Monthly averaged half-hourly data for evapotranspiration, air temperature, vapor pressure deficit and net radiation from 2009 to 2011.**
(XLS)
